# Development of a Functioning Measurement Scale for the South Korean Population Using the Korea National Health and Nutrition Examination Survey

**DOI:** 10.3389/ijph.2026.1609047

**Published:** 2026-03-09

**Authors:** Jiin Kim, Carolina Fellinghauer, Jsabel Hodel, Youngtae Cho, Wanho Kim, Carla Sabariego

**Affiliations:** 1 Faculty of Health Sciences and Medicine, Universitat Luzern, Lucerne, Switzerland; 2 National Rehabilitation Center, Seoul, Republic of Korea; 3 Schweizer Paraplegiker-Forschung, Nottwil, Switzerland; 4 Population Policy Research Center, Graduate School of Public Health, Seoul National University, Seoul, Republic of Korea; 5 Haeundae Sharing and Happiness Hospital, Busan, Republic of Korea; 6 Center for Rehabilitation in Global Health Systems, Universitat Luzern, Lucerne, Switzerland

**Keywords:** functioning, international classification of functioning, disability and health, Korean adults, national health survey, rasch model

## Abstract

**Objectives:**

This study aimed to develop a functioning metric for the Korean general population using the Korea National Health and Nutrition Examination Survey (KNHANES) and to evaluate its psychometric properties.

**Methods:**

A Partial Credit Model (PCM) calibrated functioning items from KNHANES on a 0–100 scale. Psychometric properties examined, including item fit, unidimensionality, local item dependency (LID), targeting, and differential item functioning (DIF) across age, sex, and region.

**Results:**

Using data from 5,413 adults, 14 functioning items were used to construct the metric. Model fit was achieved through a testlet solution. Item fit and unidimensionality were acceptable, with no LID observed. Reliability was adequate, though targeting indicated items were easy for the sample. Limited DIF by age was detected.

**Conclusion:**

This study developed a standardized, policy-relevant functioning metric using national health data. The developed functioning metric captures lived health and complements traditional indicators of mortality and morbidity. The study highlights the value of repurposing routine survey data for monitoring population health. Given increasing demand for health assessment in ageing societies, this metric supports evidence-based policymaking and long-term planning for Korea.

## Introduction

South Korea is one of the most rapidly ageing countries and is projected to become a super-aged society by 2026, defined as having more than 21% of the population aged 65 or older [1–3]. While people are living longer, multimorbidity has become increasingly common, affecting 54.9% of adults aged 65 and above in South Korea [4]. The most common multimorbidity pattern involves hypertension and osteoarthritis [5]. Hypertension is a major risk factor for stroke and cardiovascular events [6], whereas osteoarthritis directly affects people´s mobility, leading to withdrawal from participating in home or social activities [7]. These two conditions are important examples of the disabling impact of chronic diseases, contributing to functional decline in older adults [8, 9]. As the ageing population grows, the burden of such chronic conditions is expected to increase at the population level [5, 10].

According to the World Health Organization (WHO), health is “more than the absence of disease and infirmity” [11]. Traditional health indicators, such as mortality and morbidity, may not fully capture population health, as they overlook the severity of health conditions and their interactions with environment [11–13]. This is particularly evident in older adults, whose health reflects complex interactions among physiological changes, disease, and multimorbidity [12]. A functioning-based health indicator [14] is valuable in complementing disease-based indicators to better address the challenges of population ageing [12]. Functioning, as defined by WHO, refers to the impact of health conditions on a person’s life, encompassing biological and lived health, which includes actual performance in interaction with the environment [15–17]. Incorporating functioning-based assessments into national data systems enables the development of indicators, like disability-free life expectancy, to inform population health and ageing-related policy [18, 19].

The International Classification of Functioning, Disability and Health (ICF) [15] provides a universal framework for describing overall health and enables standardized reporting of functioning and disability [20, 21]. Based on this framework, functioning metrics have been developed using data from population surveys, such as the English Longitudinal Study of Ageing (ELSA), and the Survey of Health, Ageing and Retirement in Europe (SHARE) [22–24], applying the Rasch model, which estimates underlying functioning ability based on item difficulty and respondents’ ability [25, 26]. Recent studies have explored the feasibility of deriving functioning metrics in Korea. Kim et al. [27] applied the ICF linking rules [28]–which connect key concepts from source information to ICF categories–to identify functioning information collected in Korean health surveys. They confirmed that survey items were linkable to ICF categories and sufficiently covered key ICF domains [27]. For example, items from the Korea National Health and Nutrition Examination Survey (KNHANES) [29] were linked to six of the seven ICF Generic-7 Set categories [30], a minimal standard for assessing and reporting population functioning [27]. These findings indicate the suitability of KNHANES for population-level functioning monitoring [27].

Despite this potential, no study has yet validated a Rasch-based functioning metric in Korea. Previous studies in Korea generated only ordinal functioning scores using summative or predefined scoring methods [31, 32], without establishing a psychometrically grounded functioning scale. While Rasch-based metrics have been validated in other countries, they have predominantly focused on older adults [22, 23]. Our study extends this approach to the general adult population in Korea, enhancing its utility for population-level health assessment. For clarity, we distinguish three related terms: *metric* refers to the scale itself, *score* to an individual’s value on that scale, and *indicator* to summary statistics, such as mean or median derived from individual scores [22, 24, 33].

This study aims to develop a functioning metric for Korea’s general population using KNHANES data [29] and evaluate its psychometric properties through Rasch analysis. The resulting metric is expected to serve as a foundational resource for monitoring population health within the ICF framework and for informing public health and rehabilitation strategies, particularly in addressing the challenges posed by Korea’s transition to a super-aged society.

## Methods

### Data

We used data from the KNHANES, an annual survey producing representative statistics on the health status, health behaviors, and nutrition intake of the Korean population [34]. The KNHANES operates in three-year cycles [34]. For this study, we analyzed data from the second year (2020) of the 8th cycle (2019–2021). The survey follows a multistage clustered probability sampling design [35]. Primary sampling units (PSUs) were enumeration districts, stratified by region, urbanicity, and housing type [34]. Full methodology and datasets are available online (https://knhanes.kdca.go.kr/knhanes/main.do, Korean only) [34, 35]. While most survey items remain consistent across years within a cycle, some items are included only in specific years as part of a rotating module (Table 1). Using the standardized ICF linking rules [36], we previously identified 15 functioning-related items from the KNHANES 2020 questionnaire [27]. Those items were linked to their corresponding ICF categories [27], and were used in this study.

**TABLE 1:** International Classification of Functioning, Disability and Health (ICF)-linked Korea National Health and Nutrition Examination Survey (KNHANES) items related to functioning limitations and their availability across the 8th cycle (2019–2021). Korea National Health and Nutrition Examination Survey, Republic of Korea, 2019–2021.

ICF category	Label	Item identifier	2019	2020	2021
d230	Confirmation of activity limitation	LQ4_00	✓	✓	✓
b130, b455	Bedridden status in the past month	LQ1_sb	✓	✓	✓
d850	Confirmation of absent from school or work due to illness or impairment in the past month	LQ2_ab	✓	✓	✓
b455, d450	EQ-5D^a^: exercise ability	LQ_1EQL	✓	✓	​
d510, d540	EQ-5D: self-care	LQ_2EQL	✓	✓	​
d230	EQ-5D: daily activities	LQ_3EQL	✓	✓	​
b280	EQ-5D: pain/discomfort	LQ_4EQL	✓	✓	​
b152	EQ-5D: anxiety/depression	LQ_5EQL	✓	✓	​
d451	HINT-8^b^: climbing stairs	LQ_1HT	✓	​	✓
b280	HINT-8: pain	LQ_2HT	✓	​	✓
b130	HINT-8: energy	LQ_3HT	✓	​	✓
d850	HINT-8: work	LQ_4HT	✓	​	✓
b152	HINT-8: depression	LQ_5HT	✓	​	✓
b144	HINT-8: memory	LQ_6HT	✓	​	✓
b134	HINT-8: sleep	LQ_7HT	✓	​	✓
b134	Sleepiness	BP17_3	✓	✓	✓
d240	Feeling stress in daily life	BP1	✓	✓	✓
b130	PHQ-9^c^: little interesting or fun in doing work	BP_PHQ_1	​	✓	​
b152	PHQ-9: feeling subdued, depressed or hopeless	BP_PHQ_2	​	✓	​
b134	PHQ-9: difficulty falling asleep, waking up, or sleeping too much	BP_PHQ_3	​	✓	​
b130	PHQ-9: tiredness, low energy	BP_PHQ_4	​	✓	​
b130	PHQ-9: loss of appetite or overeating	BP_PHQ_5	​	✓	​

“Item identifier” refers to the variable name used in the KNHANES dataset. ✓ indicates the availability of each item each year.

^a^ EuroQol 5 Dimension.

^b^ Health-related Quality of Life Instrument with 8 items.

^c^ Patient Health Questionnaire-9. Labels such as EQ-5D, HINT-8, and PHQ-9 indicate the origin of certain KNHANES items and do not imply that the original instruments were directly administered or used in this study.

We applied three exclusion criteria: (1) participants under 19 years old; (2) more than 30% of missing responses across functioning items; (3) functioning items with a missing rate over 30%. The 30% cutoff was adopted to ensure measurement reliability [22, 37]. In the original KNHANES coding, scoring directions were inconsistent across items; in some items, higher scores indicated more difficulties in functioning, while in others, higher scores represented fewer difficulties. To ensure higher scores consistently reflect better functioning, reverse coding was applied to negatively worded items.

Missing responses were minimal (<0.7%) across all functioning items except BP17_3, which showed structural missingness because it was administered only to participants aged ≥40 years. Item-level mean replacement was applied to all functioning items with missing values to preserve the observed distribution.

### Statistical Analysis

#### Descriptive Statistics

Descriptive statistics included information of the study population in terms of demographic and socioeconomic characteristics such as sex, age, income, education, and marital status. The residential areas were grouped into three categories based on Korea’s administrative divisions: capital city, metropolitan city, and province. There are six metropolitan cities in Korea, with generally have higher population densities relative to their land area than the provinces. Descriptive analyses incorporated sampling weights, stratification and PSUs to account for its complex sampling design and ensure the representativeness of the national population [34].

#### Rasch Analysis

The analysis applied the Partial Credit Model (PCM), a Rasch model suitable for both dichotomous (e.g., yes or no) and polytomous (e.g., 3-or 4- ordinal categorical responses) item ratings [38]. PCM is appropriate when response categories vary across items and equal intervals cannot be assumed [38, 39]. To test the fundamental assumptions underlying Rasch modeling, the following procedures were conducted.

First, unidimensionality and local item dependencies (LID) were evaluated [40]. Unidimensionality means that all items measure a single underlying ability or trait [41]. We assessed this using principal component analysis (PCA) of Rasch residuals, with a second eigenvalue >1.4 indicating multidimensionality [42, 43]. LID refers to item dependence, where a response to one item affects another [41]. Item pairs with residual correlations >0.2 were considered locally dependent and combined into “testlets” to improve model fit [41, 44, 45].

Second, item fit was assessed using Infit and outfit mean square (MNSQ) statistics [40], with acceptable values ranging from 0.7 to 1.3 [23]. Values outside this range may indicate misfit, with those above 1.3 being particularly detrimental due to measurement noise and reduced discriminative ability [40, 46]. Additionally, threshold disordering was examined, which occurs when respondents cannot consistently distinguish between response categories, often due to unclear labels or an excessive number of options [25]. When detected, response categories were collapsed to improved model fit [25].

Third, person-fit statistics were used to detect inconsistent respondent patterns and assess data quality [46]. Reliability and measurement precision were examined using Cronbach’s alpha and the Person Separation Index (PSI), with values >0.7 indicating adequacy for group-level analysis [40, 47]. Item-person targeting was also assessed, with a difference >0.5 logits between mean item difficulty and mean person ability considered suboptimal targeting, potentially producing ceiling or floor effects [48].

Lastly, Differential Item Functioning (DIF) was examined across sex (male; female), age (19≤ years <45; 45≤ years <65; 65≤ years), and region (capital city; metropolitan city; province) [43]. DIF, indicating potential bias across groups and threatening measurement fairness [22], was evaluated using ordinal logistic regression, with McFadden’s pseudo R-squared >0.01 as the criterion [49]. Rasch analysis was conducted iteratively until acceptable model fit was achieved.

#### Functioning Metric Development

To develop the functioning metric, Rasch analysis was used to transform ordinal raw scores into an interval-scale metric ranging from 0 (extreme problems in functioning) to 100 (no problems in functioning), after confirming model fit [13, 24]. This transformation addresses the limitations of ordinal data, enabling parametric analysis and meaningful comparisons [10, 22].

Data analyses were performed using R software version 4.4.1. The eRm package was used for Rasch analysis with the PCM [50], and lordif for DIF analysis [49].

## Results

### KNHANES Sample

**FIGURE 1:**
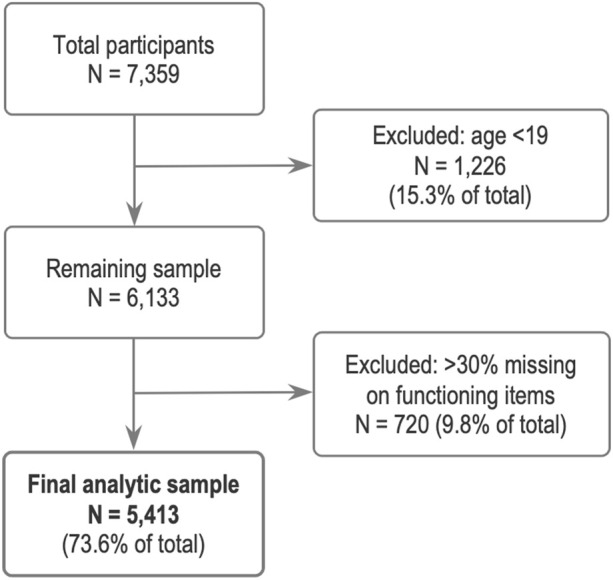
Flow chart of participant selection. Korea National Health and Nutrition Examination Survey, Republic of Korea, 2020.

A total of 7,359 individuals participated in the 2020 KNHANES. After excluding those under 19 years of age (n = 1,226) and individuals with more than 30% missing responses across the 15 functioning items (n = 720), 5,413 participants remained for analysis (Figure 1). The age range of participants was 19–80 years and over. In the KNHANES dataset, individuals aged ≥80 years were grouped together and coded as 80. Therefore, we provide the weighted median age, which was 47 years (interquartile range [IQR] = 26). Among them, 2,437 (50%, weighted) were male, and 2,279 (44.5%, weighted) resided in capital or metropolitan areas. Most participants were married and had at least a high school education. Regarding self-rated health, 4,367 (82.3%, weighted) reported their health as very good, good, or fair, while 17.7% (weighted) rated their health as poor or very poor. Descriptive statistics of the study population are shown in Table 2.

**TABLE 2:** Descriptive statistics of the study population (unweighted N = 5,413; weighted estimates representative of the national population). Korea National Health and Nutrition Examination Survey, Republic of Korea, 2020.

Characteristics	Category	Value or N (unweighted)	Value or N (weighted)	% (weighted)
Age (mean; median)		51.36; 53	47.32; 47	​
Region	Capital city (Seoul)	1,116	7,979,283	19.9
​	Metropolitan city^a^	1,163	9,887,042	24.6
​	Province^b^	3,134	22,323,766	55.5
Sex	Male	2,437	20,112,034	50
​	Female	2,976	20,078,057	50
Income	Low	1,306	9,575,698	23.9
​	Middle low	1,357	9,919,328	24.8
​	Middle high	1,365	10,342,494	25.8
​	High	1,367	10,234,820	25.5
Education	<=Elementary school	919	4,585,619	11.4
​	Middle school	523	3,056,278	7.6
​	High school	1,901	15,515,332	38.7
​	University/college=<	2,060	16,992,988	42.3
Marriage	Married	4,286	29,568,454	73.6
​	Unmarried	1,127	10,621,637	26.4
General health	Very good	254	1,969,795	4.9
​	Good	1,313	10,198,014	25.4
​	Fair	2,800	20,916,198	52
​	Poor	867	6,136,240	15.3
​	Very poor	179	969,844	2.4

Weighted values are based on survey sample weights provided by the KNHANES 2020 complex sampling design. Percentages (%) represent weighted proportions.

^a^ Metropolitan city covers an area between 500 and 1,500 km^2^ and has a population of around 1.1∼3.3 million.

^b^ Province covers an area between 7,000 and 19,000 km^2^ and has a population of around 1.5∼13 million.

Originally, 15 functioning items were selected for analysis. However, one item—“absent from school or work due to illness or impairment (d850)”—had more than 30% missing responses and was excluded. The remaining 14 items were used to build the functioning metric, and their response distributions are shown in Table 3. These items were linked to ten distinct ICF categories: five under the Body Functions component (e.g., mental functions, sensory functions, and pain), and the rest under the Activities and Participation component (e.g., self-care and mobility) [15].

**TABLE 3:** Response distribution of Korea National Health and Nutrition Examination Survey (KNHANES) items selected to build the metric of functioning. Korea National Health and Nutrition Examination Survey, Republic of Korea, 2020.

Item identifier	Label	Linked ICF code	Response option	Raw→ Modified score	N	%
BP1	Feeling stress in daily life	d240	Feel very much	1→0	26	4.9
			Feel a lot	2→1	1,226	22.7
			Feel a little	3→2	3,085	57
			Hardly feel	4→3	835	15.4
BP_PHQ_1	Little interesting or fun in doing work	b130	Almost everyday	3→0	215	4
			Over a week	2→1	121	2.2
			For several days	1→2	834	15.4
			Not at all	0→3	4,243	78.4
BP_PHQ_2	Feeling subdued, depressed or hopeless	b152	Almost everyday	3→0	121	2.2
			Over a week	2→1	119	2.2
			For several days	1→2	704	13
			Not at all	0→3	4,469	82.6
BP_PHQ_3	Difficulty falling asleep, waking up, or sleeping too much	b134	Almost everyday	3→0	470	8.7
			Over a week	2→1	242	4.5
			For several days	1→2	1,149	21.2
			Not at all	0→3	3,552	65.6
BP_PHQ_4	Tiredness, low energy	b130	almost everyday	3→0	429	7.9
			Over a week	2→1	256	4.8
			For several days	1→2	1,533	28.3
			Not at all	0→3	3,195	59
BP_PHQ_5	Loss of appetite or overeating	b130	Almost everyday	3→0	176	3.3
			Over a week	2→1	144	2.7
			For several days	1→2	652	12
			Not at all	0→3	4,441	82
BP17_3	Sleepiness	b134	Yes	1→0	1,156	21.4
			No	2→1	4,257	78.6
LQ1_sb	Bedridden status in the past month	b130, b455	Yes	1→0	300	5.5
			No	2→1	5,113	94.5
LQ_1EQL	EuroQoL: exercise ability	b455, d450	I have to lie down all day	3→0	19	0.4
			I have some problem walking	2→1	684	12.6
			I have no problem walking	1→2	4,710	87
LQ_2EQL	EuroQoL: self-care	d510, d540	I cannot take a bath or get dressed myself	3→0	8	0.1
			I have some problems taking a bath or getting dressed myself	2→1	177	3.3
			I have no problem taking a bath or getting dressed	1→2	5,228	96.6
LQ_3EQL	EuroQoL: daily activities	d230	I cannot do daily activities	3→0	19	0.4
			I have some problems doing daily activities	2→1	333	6.1
			I have no problem doing daily activities	1→2	5,061	93.5
LQ_4EQL	EuroQoL: pain/discomfort	b280	I have very severe pain or discomfort	3→0	97	1.8
			I have some pain or discomfort	2→1	996	18.4
			I have no pain or discomfort	1→2	4,320	79.8
LQ_5EQL	EuroQoL: anxiety/depression	b152	I am very anxious or depressed	3→0	32	0.6
			I am somewhat anxious or depressed	2→1	485	9
			I am not anxious or depressed	1→2	4,896	90.4
LQ4_00	Confirmation of activity limitation	d230	Yes	1→0	397	7.3
			No	2→1	5,016	92.7

Raw→Modified Score indicates that the original KNHANES response values were transformed to ensure that higher scores consistently represent greater difficulty in functioning. Items already aligned with this scoring direction were not reverse-coded, but their response categories were shifted to begin at zero.

In most items, the majority of participants reported no or only minimal difficulties in functioning. However, a few specific items showed notably lower rates of no problems in functioning. The lowest rates were observed for *BP1 - feeling stress in daily life* (15.4%), linked to d240 Handling stress and other psychological demands, and *BP_PHQ_4 - tiredness, low energy* (59%), linked to b130 Energy and drive functions. Two items (*BP_PHQ_3 and BP17_3*), linked to b134 Sleep functions, also showed relatively low rates (65.6% and 78.6%).

### Rasch Analysis

The initial Rasch analysis revealed that the data did not meet the model’s assumptions. At baseline, some of the items presented LID, and threshold disordering. The scale as a whole was multidimensional. To address these issues, we followed Baghaei’s approach [39] and created two testlets combining correlated items:

- Testlet 1 (Physical function & wellbeing): LQ_1EQL - exercise ability; LQ_2EQL - self-care; LQ_3EQL - daily activity; LQ_4EQL - pain/discomfort; LQ4_00 - activity limitation.
- Testlet 2 (Depression, subdued): LQ_5EQL - anxiety/depression; BP_PHQ_2 - feeling subdued, depressed or hopeless.

After applying testlets, the model showed no LID, supporting unidimensionality (second eigenvalue = 1.33). However, several items exhibited threshold disordering, likely due to skewed response distributions, where very few respondents selected the lowest scores (indicating severe problems in functioning) (Table 3). Accordingly, four items (*BP_PHQ_1 - little interest or fun in doing work; BP_PHQ_3 - difficulty falling asleep, waking up, or sleeping too much; BP_PHQ_4 - tiredness, low energy; BP_PHQ_5 - loss of appetite or overeating*) were initially recoded by collapsing adjacent response categories. For these items, original scores 0 and 1 were collapsed into 0, 2 into 1, and 3 into 2. This resolved disordering in BP_PHQ_1, BP_PHQ_3, and BP_PHQ_4. However, BP_PHQ_5 required further recoding into a dichotomous format (0–2 to 0; 3 to 1) to eliminate disordering.

In a subsequent step, item fit for Testlet 1 was above 1.2 and for Testlet 2 was 0.4, requiring adjustment. For Testlet 1, response categories were collapsed step by step until a dichotomous format was achieved: raw scores 0–8 were collapsed into 0 (“have a problem”) and 9 into 1 (“no problem”), improving the item fit to below 1.2. For Testlet 2, dichotomizing its original categories improved the item fit to 0.58. The detailed R code for these steps is provided in the Supplementary Material 1.

Table 4 compares psychometric properties between the initial analysis and the testlet solution (as previously described; hereafter ‘testlet solution’). With the testlet solution, Cronbach’s alpha decreased slightly from 0.79 to 0.74 but remained >0.7, indicating acceptable reliability for population surveys. In contrast, the PSI was 0.55, which may be attributed to the skewed score distribution, with most participants showing high levels of functioning. As a supplementary analysis, we used weighted sampling by 20-point intervals of functioning score (N = 120). This yielded a PSI of 0.81, suggesting the lower value in the full sample may reflect score skewness.

**TABLE 4:** Comparison of Partial Credit Model (PCM) fit statistics and reliability between the initial analysis and the testlet solution. Korea National Health and Nutrition Examination Survey, Republic of Korea, 2020.

Statistic	Initial analysis	Testlet solution
Item difficulties - mean (SD)	−0.21 (1.43)	−0.01 (1.21)
Person abilities - mean (SD)	2.51 (1.39)	1.80 (1.47)
Person separation index (PSI)	0.55	0.55
Cronbach’s alpha	0.79	0.74

SD, Standard Deviation.

In the testlet solution, the mean person ability estimate decreased from 2.51 to 1.80, bringing it closer to the mean item difficulty (from −0.21 to −0.01). This indicates better alignment between person abilities and item difficulties. However, as the discrepancy still >0.5 logits, the items appeared generally too easy for the sample, implying the scale may be better suited to assessing individuals with higher disability levels than those in this sample. We conducted additional analysis to examine whether person-item alignment differed by age group. However, no significant differences in targeting were found across age groups.

The fit statistics of the testlet solution are shown in Table 5. Four items (BP_PHQ_1, BP_PHQ_4, BP_PHQ_5, and Testlet 2) had outfit values <0.7, indicating overfit. This suggests that these items/testlet predicted response patterns too well. All other items demonstrated acceptable infit and outfit MNSQ values within the commonly recommended range (0.7–1.3). Regarding item difficulty, *BP1 - feeling stress in daily life* had the highest value (0.97), which indicated that respondents found it hardest to endorse this item. In other words, most individuals, regardless of their level of functioning, were more likely to report stress in daily life than other items. Conversely, *LQ1_sb - bedridden status in the past month* had the lowest difficulty (−1.77), meaning that most respondents did not report a bedridden status in the past month. BP1 also showed the widest measurement range (−1.14–3.60) among all items. DIF analysis revealed no notable differences by sex or region, but age-related DIF was found in two items: *BP17_3 - sleepiness* and *Testlet 1 - physical function & wellbeing*. The presence of DIF suggests that these items may function differently across age groups.

**TABLE 5:** Detailed fit statistics of tested Partial Credit Model (PCM) including item location, outfit, infit, local item dependency (LID), and differential item functioning (DIF) for initial analysis and testlet solution. Korea National Health and Nutrition Examination Survey, Republic of Korea, 2020.

Analysis type	Item identifier	Label	Item difficulty	Thresholds		Disordered thresholds	Outfit MSQ	Infit MSQ	LID	DIF
			Location	1	2					
Initial analysis	BP1	Feeling stress in daily life	1.70	−0.32	4.29	​	0.98	1.01	​	​
	BP_PHQ_1	Little interest or fun in doing work	0.39	−0.63	1.31	Yes	0.72	1.00	​	​
	BP_PHQ_2	Feeling subdued, depressed or hopeless	0.08	−0.58	0.60	Yes	0.48	0.77	​	​
	BP_PHQ_3	Difficulty falling asleep, waking up, or sleeping too much	0.95	−0.02	1.67	Yes	0.85	0.96	​	​
	BP_PHQ_4	Tiredness, low energy	1.00	−0.22	1.68	Yes	0.66	0.78	​	​
	BP_PHQ_5	Loss of appetite or overeating	0.26	−0.25	0.87	Yes	0.70	0.92	​	​
	BP17_3	Sleepiness	0.74	0.74	​	​	1.07	1.08	​	Age
	LQ1_sb	Bedridden status in the past month	−1.02	−1.02	​	​	0.82	0.94	​	​
	LQ_1EQL	Exercise ability	−1.33	−2.68	0.01	​	1.04	1.01	LQ_2EQL, LQ_3EQL, LQ_4EQL	Age
	LQ_2EQL	Self-care	−2.01	−2.43	−1.60	​	0.66	0.92	LQ_1EQL, LQ_3EQL, LQ_4EQL	Age
	LQ_3EQL	Daily activities	−1.48	−2.08	−0.88	​	0.58	0.88	LQ_1EQL, LQ_2EQL, LQ_4EQL	Age
	LQ_4EQL	Pain/discomfort	−0.34	−1.25	0.58	​	0.90	0.98	LQ_1EQL, LQ_2EQL, LQ_3EQL	Age
	LQ_5EQL	Anxiety/depression	−1.14	−1.85	−0.42	​	0.51	0.81	​	​
	LQ4_00	Activity limitation	−0.69	−0.69	​	​	0.71	0.91	LQ_3EQL	Age
Testlet solution	BP1	Feeling stress in daily life	0.97	−1.14	3.60	​	0.92	0.94	​	​
	BP17_3	Sleepiness	0.03	0.03	​	​	1.11	1.13	​	Age
	LQ1_sb	Bedridden status in the past month	−1.77	−1.77	​	​	0.86	0.92	​	​
	BP_PHQ_1	Little interest or fun in doing work	−0.36	−0.52	−0.21	​	0.65	0.87	​	​
	BP_PHQ_3	Difficulty falling asleep, waking up, or sleeping too much	0.35	0.19	0.52	​	0.77	0.88	​	​
	BP_PHQ_4	Tiredness, low energy	0.45	−0.09	1.00	​	0.61	0.71	​	​
	BP_PHQ_5	Loss of appetite or overeating	−0.24	−0.24	​	​	0.67	0.85	​	​
	Testlet 1	Physical function & wellbeing	0.44	0.44	​	​	1.18	1.14	​	Age
	Testlet 2	Depression, subdued	−0.02	−0.02	​	​	0.58	0.79	​	​

**FIGURE 2:**
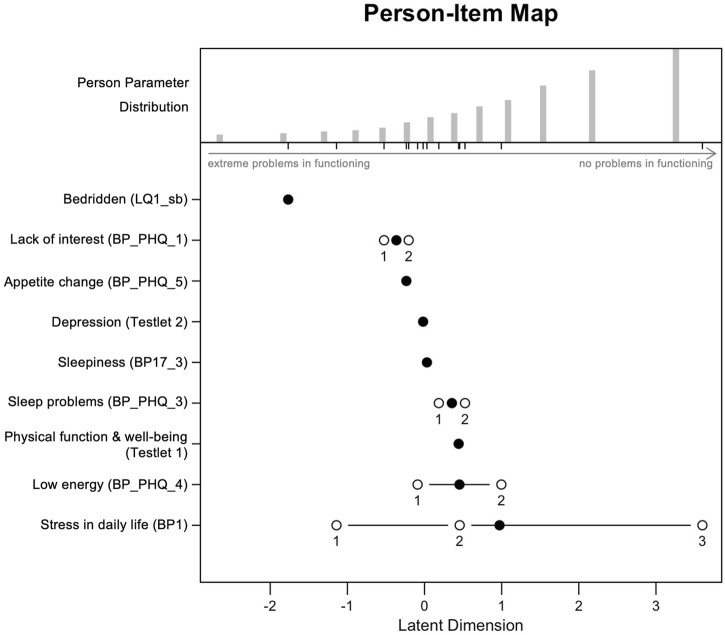
Person-Item Map for the final Partial Credit Model (testlet solution), showing the distribution of person abilities and item difficulties along the latent scale (functioning score). Korea National Health and Nutrition Examination Survey, Republic of Korea, 2020.

The results of the testlet solution are visualized in the Person-Item Map (Figure 2), showing the distribution of person abilities and item difficulties along the latent scale (functioning score). The upper section shows person parameters, indicating the relative ability levels of respondents (i.e., their levels of functioning), with individuals at higher levels of functioning appearing further to the right. The lower section shows item locations along the same latent scale, reflecting item difficulty (i.e., how likely respondents were to report no problems on that item), with items located further to the right representing more difficult items (e.g., *BP1*), and those on the left indicating easier items (e.g., *LQ1_sb*). Item difficulties are indicated by black dots, and threshold locations between response categories by white dots. The distribution of person abilities is slightly right-skewed compared to item difficulties, suggesting a potential ceiling effect (i.e., the metric assesses levels of disability above what is found in the population).

Table 6 presents the transformation of raw scores into a user-friendly functioning scores ranging from 0 (extreme problems in functioning) to 100 (no problems in functioning). In the initial analysis, the maximum total raw score was 31, as described in Table 2. However, in the testlet solution, response categories were collapsed, resulting in a total score ranging from 0 to 14.

**TABLE 6:** Transformation table showing the raw scores and corresponding interval-scaled 1–100 functioning scores. Korea National Health and Nutrition Examination Survey, Republic of Korea, 2020.

Raw total score	Logit score (PCM analysis)	0–100 functioning score
0	−3.55	0.00
1	−2.65	11.20
2	−1.83	21.51
3	−1.30	28.10
4	−0.89	33.20
5	−0.55	37.55
6	−0.23	41.52
7	0.08	45.33
8	0.39	49.17
9	0.71	53.26
10	1.08	57.89
11	1.54	63.57
12	2.17	71.50
13	3.26	85.07
14	4.45	100.00

Raw total score is developed after collapsing response options.

## Discussion

This study developed a functioning metric for the Korean population using the KNHANES data, and validated it through the Rasch model. Model fit was achieved through a testlet solution and response category collapsing, ensuring that the final model satisfied the key assumptions of the PCM. Some items showed outfit MNSQ values <0.7, suggesting potential overfit [40]. However, unlike underfit, overfit rarely affects measurement quality in practice and is not considered a violation of psychometric properties [40]. The final functioning metric was transformed to a 0–100 scale to provide a standardized, policy-relevant measure of health of the general population, conceptually aligned with the ICF framework [15]. This functioning metric and the resulting score can be used for building a functioning indicator to synthesize the general level of health in a population, a measure often overlooked by the traditional health indicators, such as mortality and morbidity, thereby offering complementary perspective on population health [14].

This work is in line with prior studies aiming to develop a functioning metric using population datasets, for example, by Oberhauser et al. [23] and Fellinghauer et al. [22]. While methodologically aligned, our study differs in its application to a nationally representative adult population, using a single year of KNHANES data and incorporating all available functioning items. The final metric included data from ten distinct ICF categories, all of which are part of the Generic-30 Set [51]—a minimal set of 30 categories for assessing functioning and disability in both clinical and population health contexts—underscoring their relevance in evaluating core aspects of functioning. Although our analysis focused on a single time point, KNHANES’s repeated annual structure [34] offers potential for multi-year metric development. Similarly, Caballero et al. [52] developed a longitudinal functioning metric using multiple years of ELSA data, based on 39 anchor items.

Our study highlights the practical value of repurposing existing national health datasets to derive meaningful insights into population health using the comprehensive operationalization of functioning proposed by WHO [16, 53]. Given the increasing demand for health monitoring in ageing societies [4], maximizing the utility of routinely collected national data offers a cost-effective alternative to developing new assessment tools and collecting additional data, which require substantial time and resources [54]. KNHANES has been annually implemented since 2007 [34, 35], offering a solid basis for monitoring population-level trends in functioning over time. Its breadth spanning health conditions, health behaviors, healthcare utilization, and clinical indicators [34] also facilitates analyses of relationships between health-determinants and functioning.

The developed functioning metric is valid for population-level use, though the refinement of data collection aspects would improve its psychometric properties. Potential future opportunities for refinements are relatively minor. A key issue lies in the skewed distribution of functioning ability. For instance, the scarcity of responses indicating severe limitations in functioning led to the collapsing of response categories to improve model fit, thereby reducing data granularity [39]. To address underrepresentation of individuals with lower functioning, incorporating data from the Korean Longitudinal Healthy Ageing Cohort (KLHAC) [55] could improve understanding of high-risk subpopulations [17]. KLHAC can be linked to data from the National Health Insurance Service (NHIS), which include medical and long-term care records, and potentially aligned with the ICF framework [4, 55]. A second opportunity is to integrate ICF-based surveys (e.g., the brief version of the Model Disability Survey (MDS) [56]) into existing national surveys to increase the specificity and comprehensiveness of functioning data [17, 24]. KNHANES offers extensive information, but it is primarily disease-focused. Integrating instruments such as the brief MDS could provide more refined insights into functioning and support international comparability. Finally, item-level improvements are also warranted. We observed variation in threshold spacing across items: for example, *BP1 - feeling stress in daily life* showed noticeably wide intervals. In such cases, additional response categories could improve measurement precision [57].

This functioning metric has practical implications for public health strategies, particularly in the context of multimorbidity and population ageing. In Korea, although healthy life expectancy (HALE) is high, healthy ageing score remains relatively low [58], suggesting a mismatch between disease-based indicators and lived health. This discrepancy highlights the need for functioning-based indicators in population health monitoring. As shown in our study, a functioning metric utilizing the ICF as a universal reference enables standardized reporting and allows for quantitative group comparisons on an interval scale, rather than the commonly used ordinal scales [17]. Moreover, the routine use of functioning information can support a learning health system (LHS), strengthening a continuous cycle that integrates research, policy, and practice based on sound scientific evidence [17, 59]. The metric therefore has potential utility in national surveillance, rehabilitation planning, and long-term care policy development.

In Korea, prior studies have measured functioning using the ICF framework, for example, in stroke or breast cancer [60, 61], but these have largely focused on specific conditions. To our knowledge, no study has validated psychometric properties of a functioning metric using a nationally representative dataset of the entire population. An additional noteworthy finding is that, contrary to expectations that older adults would report lower levels of functioning, in some items—such as *BP1 - feeling stress in daily life, BP_PHQ_1 - little interesting or fun in doing work,* and *BP_PHQ_4 - tiredness, low energy*—a higher proportion of older adults (aged 60 and above) reported ‘no problems’ compared to the younger group (aged 19–44). This may reflect the greater mental health burden among younger generations in Korea, as well as broader social factors such as intergenerational economic disparities [62, 63]. This observation suggests that functioning patterns need to be interpreted in light of the country-specific sociocultural context, rather than solely through age-based assumptions.

### Limitations

This study has several limitations. First, LID was detected among some functioning items, necessitating the construction of testlets. This aggregation of multiple ordinal items increased the number of response categories, which appeared to contribute to threshold disordering [24]. Accordingly, response categories were dichotomized, resulting in a certain degree of information loss [39], which may reduce the precision of measurement. Second, items *LQ_1EQL - exercise ability, LQ_2EQL - self-care*, and *LQ_3EQL - daily activities* showed extremely low response frequencies for severe problems in functioning. Such irregular distributions are common in clinical or population data, as many respondents tend to report good functioning [40]. However, infrequently used response categories may also indicate unnecessary or redundant distinctions between categories, or suboptimal item wording or content [23, 40]. Moreover, sparse endorsement in extreme categories may limit the sensitivity of these items in detecting differences among individuals with lower levels of functioning. Third, DIF was observed across age groups, particularly for item related to sleepiness and physical function & wellbeing, which is plausible given that fatigue and mobility limitations generally increase with age. This DIF may reflect true age-related variation rather than measurement bias [22, 24]. However, it still suggests that the comparability of scores across age groups should be interpreted with caution. Lastly, because the metric was developed using nationally collected data, its application may be context-specific. Nevertheless, it remains conceptually aligned with the internationally recognized ICF framework, as it was constructed using items previously linked to ICF categories. Future studies are needed to evaluate its applicability across different population contexts.

### Conclusion

This study developed a standardized, policy-relevant functioning metric for the Korean general population using national health data. By capturing lived health, the metric can generate a functioning indicator that complements the traditional health indicators of mortality and morbidity. The study highlights the value of repurposing routine survey data for monitoring population health. With growing demand for health assessment in ageing societies, this approach offers a sustainable strategy that strengthens international comparability and supports evidence-informed policy on population functioning.

## Data Availability

The data used in this study were obtained from the Korea National Health and Nutrition Examination Survey (KNHANES), an open and publicly accessible national survey. The dataset is available from the Korea Disease Control and Prevention Agency (KDCA) website (https://knhanes.kdca.go.kr/knhanes/main.do, accessible via “Raw data > Download”; the download interface is available only in Korean) upon request following their data access procedures. As the data are publicly available, no additional data repository submission is required.
