# Supplementary material for: Development of a Functioning Measurement Scale for the South Korean Population Using the Korea National Health and Nutrition Examination Survey
Source: Int J Public Health. 2026 Mar 9;71:1609047. doi: 10.3389/ijph.2026.1609047 (PMC13006414; doi:10.3389/ijph.2026.1609047)
Supplement: Supplementary file 1 [file Supplementaryfile1.docx]

**International Journal of Public Health**

**Title:** Developing a functioning metric for the Korean general population using national health survey data

**Supplementary File 1. Data preparation before Partial Credit Model (PCM) analysis**

The exclusion criteria applied to derive the analytic sample are summarized in Figure 1 (main text). In practice, these exclusions were implemented during the data preparation stage by applying filters to the raw dataset. The key steps were as follows:

1. Participants younger than 19 years were removed.

2. Participants with missing responses on more than 30% of the 15 functioning items were removed.

After exclusions, the final analytic sample size was N = 5413, as reported in the main text.

**Data preparation and recoding steps (R code)**

Before conducting the Partial Credit Model (PCM) analysis, the dataset was further prepared as follows:

1. Items showing local item dependency (LID) were combined into testlets

2. Items with threshold disordering or poor fit were recoded by collapsing response categories.

The final analytic dataset (data_reduced_4) was created for input into the PCM analysis.

The R code below documents these preparation steps:

| library(readxl)  #Load dataset  knhanes <- read_excel("data.xlsx")  #Variables for analysis  var1 = c("BP1", "BP_PHQ_1", "BP_PHQ_2", "BP_PHQ_3", "BP_PHQ_4", "BP_PHQ_5", "BP17_3", "LQ1_sb", "LQ_1EQL", "LQ_2EQL",  "LQ_3EQL", "LQ_4EQL", "LQ_5EQL", "LQ4_00")  pf = c("year", "region", "sex", "age", "incm", "edu", "marri_1", "D_1_1")  id = "ID"  data = knhanes[, c(id, pf, var1)]  #Add dummy cases to cover full score range  dummy.min = rep(0, length(var1))  dummy.max = apply(data[,var1], 2, max)  data.PCM = rbind(data[, var1], dummy1 = dummy.min, dummy2 = dummy.max)  #Composite items  data.PCM$testlet1 <- rowSums(data.PCM[, c("LQ_1EQL", "LQ_2EQL",  "LQ_3EQL", "LQ_4EQL", "LQ4_00")],  na.rm = TRUE)  data.PCM$testlet2 <- rowSums(data.PCM[, c("BP_PHQ_2", "LQ_5EQL")],  na.rm = TRUE)  #Remove original items used in composites  data_reduced <- data.PCM[, !(names(data.PCM) %in%  c("LQ_1EQL", "LQ_2EQL",  "LQ_3EQL", "LQ_4EQL", "LQ4_00", "BP_PHQ_2", "LQ_5EQL"))]  #Recode BP_PHQ_1,3,4 (0,1->0; 2->1; 3->2), BP_PHQ_5 (0,1,2->0, 3->1)  data_reduced$BP_PHQ_1_transformed <- ifelse(data_reduced$BP_PHQ_1 %in% c(0,1),0,  ifelse(data_reduced$BP_PHQ_1 ==2,1,2))  data_reduced$BP_PHQ_3_transformed <- ifelse(data_reduced$BP_PHQ_3 %in% c(0,1),0,  ifelse(data_reduced$BP_PHQ_3 ==2,1,2))  data_reduced$BP_PHQ_4_transformed <- ifelse(data_reduced$BP_PHQ_4 %in% c(0,1),0,  ifelse(data_reduced$BP_PHQ_4 ==2,1,2))  data_reduced$BP_PHQ_5_transformed <- ifelse(data_reduced$BP_PHQ_5 %in% c(0,1,2),0,  ifelse(data_reduced$BP_PHQ_5 ==3,1,NA))  #Remove original BP_PHQ_1,3,4,5  data_reduced_2 <- data_reduced[, !(names(data_reduced) %in%  c("BP_PHQ_1", "BP_PHQ_3",  "BP_PHQ_4", "BP_PHQ_5"))]  #Recode testlet1 (0,1,2,3,4,5,6,7,8->0; 9->1)  data_reduced_2$testlet1_trans <- cut(data_reduced_2$testlet1,  breaks = c(-Inf, 8, 9, Inf),  labels = c(0, 1, 2),  right = TRUE)  data_reduced_2$testlet1_trans <-as.numeric(as.character(data_reduced_2$testlet1_trans))  #Remove original testlet1  data_reduced_3 <- data_reduced_2[, !(names(data_reduced_2) %in%  c("testlet1"))]  #Recode testlet2 (0,1,2,3,4->0; 5->1)  data_reduced_3$testlet2_trans <- ifelse(data_reduced_3$testlet2 %in% c(0,1,2,3,4),0,  ifelse(data_reduced_3$testlet2 ==5,1,NA))  #Remove original testlet2  data_reduced_4 <- data_reduced_3[, !(names(data_reduced_3) %in%  c("testlet2"))]  #Final dataset for Rasch PCM analysis  summary(data_reduced_4) |
| --- |
